# Analysis of Violent Incidents at Five Regional and Remote Australian Emergency Departments: A Retrospective Descriptive Study

**DOI:** 10.1177/23779608241261597

**Published:** 2024-07-23

**Authors:** Brodie Thomas, Alycia Jacob, Damhnat McCann, Penny Buykx, Rebecca Schultz, Leigh Kinsman, Peter O’Meara, Kristina Edvardsson, Evelien Spelten

**Affiliations:** 1 La Trobe Rural Health School, 2080 La Trobe University, Mildura, Australia; 2 School of Nursing, Midwifery and Paramedicine, 95359 Australian Catholic University, Fitzroy, Australia; 3 School of Nursing, 3925 University of Tasmania, Launceston, Australia; 4 School of Humanities, Creative Industries and Social Sciences, 5982 The University of Newcastle, Callaghan, Australia; 5 Northern Territory Health, Alice Springs, Australia; 6 Mid North Coast Local Health District, Port Macquarie, Australia; 7 Violet Vines Marshman Centre for Rural Health Research, La Trobe University, Australia; 8 Department of Paramedicine, 2541 Monash University, Frankston, Australia; 9 School of Nursing and Midwifery, 2080 La Trobe University, Bundoora, Australia

**Keywords:** workplace violence, emergency service, hospital, risk management, occupational injuries, security alert, code black

## Abstract

**Introduction:**

Workplace violence is endemic, destructive, and escalating in frequency and severity in healthcare. There is a paucity of research on workplace violence in regional and remote hospital emergency departments (EDs).

**Objective:**

The aim of this study was to identify the perpetrator and situational characteristics associated with violent incidents in the ED across five regional and remote Australian sites.

**Method:**

This study audited hospital summary data, incident reports, and medical records for a 12-month period in 2018 to examine the perpetrator and situational characteristics of workplace violence incidents in five regional and remote Australian EDs.

**Results:**

Violent incidents were evenly spread throughout the week and across shifts. Most incidents were triaged as urgent, occurred within the first 4 hr, and had multidisciplinary involvement. Almost one in every six incidents resulted in an injury. Perpetrators of violence were predominantly young and middle-aged males and almost always patients, with most presenting with mental and behavioral disorders, or psychoactive substance use.

**Conclusions:**

Understanding the characteristics of perpetrators of violence can help in seeking to tailor interventions to reduce further violent behaviors. These findings carry implications for optimizing patient care, staff safety and resource management.

## Background

Hospital emergency departments (EDs) can be highly stressful and challenging environments. ED staff work with individuals in a physiological or psychological crisis, while contending with demanding time constraints and crowding (Morley et al., 2018; Nikathil et al., 2017; Spelten et al., 2020b). Workplace violence, particularly violence perpetrated by patients and family members toward staff, is commonplace in this environment and has been reported to be increasing in frequency and severity internationally (Kleissl-Muir et al., 2019; Longo & Phillips, 2016; Nikathil et al., 2017). Studies conducted in Australia and internationally show workplace violence is one of the leading causes of workplace mortality and morbidity, and can have profound consequences on individuals as well as organizations and societies (CDC, 2006; Longo & Phillips, 2016; Maguire et al., 2017; Spelten et al., 2020a; Thomas et al., 2020).

Violence is a general term that can be broadened or narrowed depending on the context where it is sought to be used (De Haan, 2008). The World Health Organization Violence Prevention Alliance defines violence as:

*the intentional use of physical force or power, threatened or actual, against oneself, another person, or against a group or community, that either results in or has a high likelihood of resulting in injury, death, psychological harm, maldevelopment, or deprivation.* (WHO, 2021, p. 1)

In Australia, health departments tend to describe violence more narrowly, focusing on “incidents,” with the accepted definition of

*any incident, in which an individual is abused, threatened or assaulted*. (NSW Ministry of Health, 2015)

## Review of Literature

Violence in healthcare is a wicked problem (Jacob et al., 2022). Internationally, there have been many interventions implemented to prevent and reduce workplace violence in healthcare. The majority of these focus on education and training for healthcare workers (Ramacciati et al., 2016; Spelten et al., 2020b). There has, however, been no reliable evidence that any interventions have been effective in reducing violence in emergency healthcare (Spelten et al., 2020a). Alongside this dearth of evidence regarding effective interventions, there are increasing calls to reconsider the focus of intervention efforts: for example, shifting the focus from placing the burden of violence prevention on workers, to interventions that include a focus on perpetrators. This may provide a more comprehensive and effective approach to violence prevention (Nikathil et al., 2018; Spelten et al., 2020a, 2020b; Thomas et al., 2020). Such a shift in focus would require an understanding of the perpetrators in order for violence prevention efforts to be well-targeted.

Regional areas in Australia experience inequitable access to healthcare services compared to metropolitan areas (Australian Institute of Health Welfare, 2019b). Reduced access to healthcare in conjunction with generally lower health status has resulted in high utilization of Australian regional EDs, including higher rates of nonurgent presentations and potentially preventable hospitalizations compared to metropolitan settings (Australian Institute of Health Welfare, 2019b; Unwin et al., 2020). These EDs have more pronounced health workforce shortages, with the percentage of health professionals per population decreasing as remoteness increases (Australian College of Nursing, 2018; Australian Institute of Health Welfare, 2019b). Risky alcohol consumption and alcohol-related injuries presenting to EDs are higher in regional and rural areas compared to metropolitan centers (Australian Institute of Health Welfare, 2019a; Coomber et al., 2013; Kleissl-Muir et al., 2018). These risk factors are all associated with increased violence in healthcare settings (Al-Qadi, 2021; Nowrouzi-Kia et al., 2019).

There is not a clear understanding of the epidemiology of workplace violence in the ED due to a lack of empirical data (Nikathil et al., 2017, 2018). Utilization of inadequate data collection tools, underreporting and “glossing over” of violent incidents are common contributors to the lack of information (Nevels et al., 2020; Nikathil et al., 2018) and there is little evidence from regional areas (Kleissl-Muir et al., 2018, 2019; Nikathil et al., 2018). Most studies investigating characteristics of workplace violence use staff surveys that focus on staff perceptions rather than objective incident data (Aljohani et al., 2021; Nikathil et al., 2017). These staff perceptions indicate that alcohol and drug intoxication and mental health issues are the most common factors associated with violence in EDs (Doyle, 2015; Nikathil et al., 2018). Studies that analyze objective hospital data mostly report only one source of data, generally incident reports or security records. Incident reports alone do not portray the full complexity of violent situations as the reports provide limited insight into the perpetrator characteristics of violence and the complexity of incident reporting systems decreases the quality of reports (Kleissl-Muir et al., 2018; Nikathil et al., 2017; Thomas et al., 2021). Few studies have analyzed incident reports with patient medical records to provide an in-depth understanding of the characteristics of perpetrators (Kleissl-Muir et al., 2018; Nikathil et al., 2017). Studies that include hospital records consistently report that verbal violence is the most common type of violence (Aljohani et al., 2021; Kleissl-Muir et al., 2019; Liu et al., 2019; Nikathil et al., 2017), males are the predominant perpetrators (Department of Human Services, 2005; Downes et al., 2009; Knott et al., 2005; Nikathil et al., 2017, 2018), and the most common precipitant to violence is psychoactive substance use (Department of Human Services, 2005; Downes et al., 2009; James et al., 2006; Kleissl-Muir et al., 2018, 2019; Knott et al., 2005; Nikathil et al., 2017, 2018). Mental health issues are commonly yet inconclusively reported as precipitants to violence, with rates of associated mental health issues ranging from 14% to 78% of incidents (Kleissl-Muir et al., 2018; Knott et al., 2005; Nikathil et al., 2017, 2018). Data from regional and remote EDs in Australia, utilizing both incident reports and medical records is required to gain an understanding of the complex characteristics of workplace violence in the ED and to contribute to more targeted efforts to reduce violence (Aljohani et al., 2021; Kleissl-Muir et al., 2018; Liu et al., 2019; Nikathil et al., 2017).

## Objectives of the Study

The aim of this study was to identify the perpetrator and situational characteristics associated with violent incidents in the ED across five regional and remote Australian sites.

## Method

### Design

This is a retrospective descriptive study of violent incidents based on incident or security reports and patient medical records from five regional and remote Australian EDs over a 12-month period (01/01/2018 to 31/12/2018). The five sites were included to portray a diverse cross-section of regional and remote experience. Researchers from each of these sites collaborated to share information and context-specific learnings.

#### Definition of Security Alerts

As the EDs were located across different jurisdictions there were some discrepancies with terminology used for reporting data. In one state, security alerts are either Code Black or Code Grey; Code black is a police and security response to an armed threat while a Code Grey is an organizational-wide clinical and security response to actual or perceived violence or aggression (Morley et al., 2018; Ramacciati et al., 2016). All other states and territories involved in this study use Code Black as an overarching security alert for urgent assistance when staff believe their safety or the safety of patients or others is at risk (SA Health, 2017).

#### Data Collection

Retrospective audits of security alerts occurring over 12 months between January 2018 and January 2019 were conducted independently across the five EDs. Data related to the situational characteristics of the security alerts were extracted from incident reports generated through incident management systems at all sites. Once incidents were identified, data related to the characteristics of people triggering security alerts (perpetrators) were collected from electronic (Sites 1, 3, and 4) and paper-based (Site 2) medical records. Only data from incident reports were available from Site 5. The triangulation of both sources of data was performed to provide the most reliable description of violent incidents. The data extraction tool was developed through consultation with nurses working in a regional ED and a review of data collection tools used in previously published studies (James et al., 2006; Knott et al., 2005). The tool was piloted at a regional hospital in 2017.

Situational characteristics extracted included: the reason for ED presentation, time and day of presentation, length of time in the ED from initial triage to time of triggering a security alert, the reason for a security alert, weapons used, people involved (including staff), injuries sustained by staff and incident response. A consistent definition of “injury” was not used due to multiple incident management systems, with each system having a specific section where injuries were recorded. Individual perpetrator characteristics extracted included age, sex, presenting condition, triage category, past history of violent behavior at that facility, alcohol and/or drug use and presenting diagnosis.

## Research Questions

What are the situational characteristics associated with violent incidents in the ED?

What are the perpetrator characteristics associated with violent incidents in the ED?

## Sample

The sample of EDs was within the resources of the research team. While there were no strict inclusion criteria, sites were not considered for inclusion if they were located within a metropolitan area, if they were specialty EDs such as children's hospitals, or if they saw less than 5,000 patients per year. The included EDs were: two EDs within a regional inland health service providing services to a population of approximately 320,000 people over 50,000 square kilometers; one ED located in a costal retirement region within a health service district providing services to a population of more than 100,000 people over 3,700 square kilometers; one ED in a remote inland health service providing services to a population of approximately 50,000 over more than a million square kilometers; and one ED in a major regional center serving an area of 20,000 square kilometers with a population of approximately 150,000.

## Ethics

Ethical approval was obtained separately for each of the sites: Bendigo Health Care Group Human Research and Ethics Committee (HREC)—LNR/17/BHCG/55 (Sites 1 and 2); North Coast NSW HREC—2020/ETH00118 HREA280 (Site 3); Central Australian HREC—CA-20-3775 (Site 4); and Tasmanian Health and Medical HREC—H0018514 (Site 5).

All methods were carried out in accordance with approved guidelines and regulations. Written consent to access data was provided by the director of the ED at each hospital. The requirement for informed consent to access individuals’ medical records was waived by the Human Research Ethics Committee at each site.

## Data Analysis

Data were entered into Excel spreadsheets at each site. The deidentified data from each site were then exported into an SPSS (IBM Corp, 2019) study database. We used descriptive statistics to summarize the situational and perpetrator characteristics associated with violent incidents. Missing data were treated as missing completely at random. Data regarding patient presenting problems, diagnoses and narrative accounts in patient notes and incident reports were used to identify the prespecified categories of perpetrators. The categories of perpetrators were chosen based on data from ED nurses and paramedics, who describe how they differentiate between different types of violent perpetrators and alter their management accordingly. These categories are based on the perceived factors associated with violent behavior and include perpetrators who have (a) no medical problem; (b) physical health issue; (c) mental or behavioral disorders (MBD) which can be related to (3a) psychoactive substance use (PSU) or; (3b) MBD other than PSU; or (3c) a complexity of issues; and (4) repeat perpetrators (Spelten et al., 2020b). Perpetrators were considered repeat perpetrators if they were responsible for security alerts over multiple presentations or if an alert for previous violent behavior was recorded. Multiple security alerts during a single presentation were counted as a single event.

## Results

**Figure 1.**
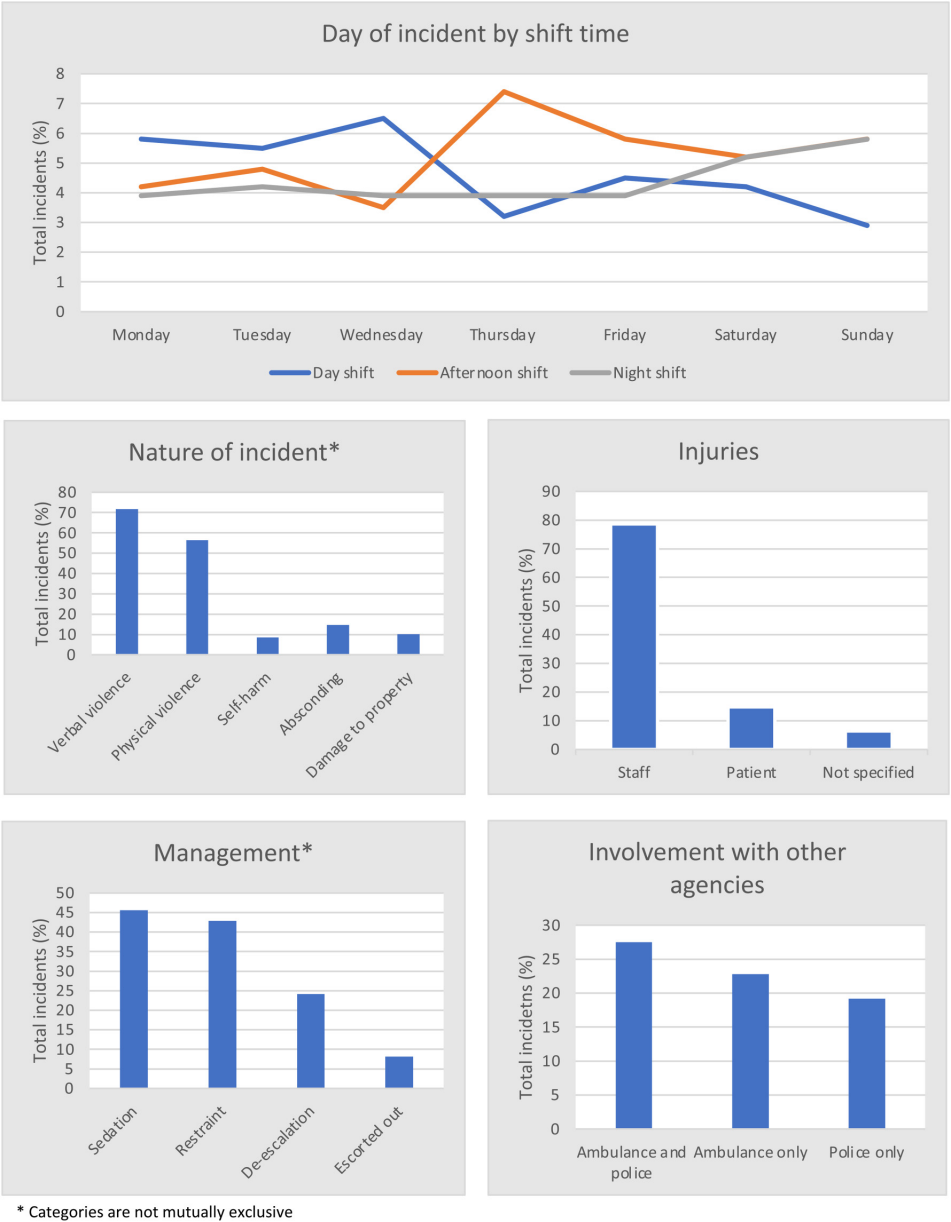
Situational characteristics of violent incidents in regional and remote Australian EDs.

*Note.* ED = emergency department.

A total of 314 violent incidents across the five EDs were identified. The number of incidents reported per site ranged from 26 to 133 (*Mdn* 79.5) with security alerts per 1000 presentations ranging from 0.48 to 2.94. Situational characteristics including day of the week, time of arrival at the ED, nature of incident and injury occurrence, management of incidents and involvement of other health and emergency services are presented in Figure 1.

Incidents were spread across the days of the week and were more likely to occur on a late shift. Many of the incidents had multiagency interaction with half of the perpetrators arriving via ambulance and just under half involving police. The majority of incidents occurred within the first four hours following triage, and most patients were triaged as Category 3 (urgent). Management of incidents included deescalation, physical restraint, sedation, and removal of perpetrators. A more comprehensive record of the situational characteristics separated across all sites is in Table 1.

**Table 1.** Situational Characteristics Summary.

	Site 1 *n* (%)	Site 2 *n* (%)	Site 3 *n* (%)	Site 4 *n* (%)	Site 5 *n* (%)	Total, *n* (%)
Total incidents	26	64	60	31	133	314*
Total patient presentations	53,768	34,012	36,018	45,649	45,170	214,617
Security alerts per 1,000 presentations	0.48	1.88	1.67	0.68	2.94	1.46
Day of week	26	64	60	31	133	314
Monday–Thursday	14 (53.8)	31 (48.4)	36 (60.0)	20 (64.5)	76 (57.1)	177 (56.4)
Friday–Sunday	12 (46.2)	33 (51.6)	24 (40.0)	11 (35.5)	57 (42.9)	137 (43.6)
Time	23	64	59	31	133	310
Early (07:00–15:30)	8 (34.8)	15 (23.4)	21 (35.6)	6 (19.4)	51 (38.3)	101 (32.6)
Late (15:30–22:00)	11 (47.8)	30 (46.9)	17 (28.8)	7 (22.6)	49 (36.8)	114 (36.8)
Night (22:00–07:00)	4 (17.4)	19 (29.7)	21 (35.6)	18 (58.1)	33 (24.8)	95 (30.6)
Location of event	13	56	60	30		159
Triage and waiting room	7 (53.8)	24 (42.9)	14 (23.3)	9 (30.0)		54 (34.0)
Emergency resus bay	3 (23.1)	3 (5.4)	2 (3.3)	2 (6.7)		10 (6.3)
Main ED cubicle	0	20 (35.7)	34 (56.7)	17 (56.7)		71 (44.7)
Other	3 (23.1)	9 (16.1)	10 (16.7)	2 (6.7)		24 (15.1)
Type of perpetrator	22	58	58	31	133	302
Patient	22 (100.0)	58 (100.0)	58 (100.0)	29 (93.5)	130 (97.7)	297 (98.3)
Bystander	0	0	0	2 (6.5)	3 (2.3)	5 (1.7)
Nature of incident**	26	62	60	29		177
Verbal violence	16 (61.5)	48 (77.4)	40 (66.7)	20 (69.0)		124 (71.8)
Physical violence	11 (42.3)	31 (50.0)	40 (66.7)	18 (62.1)		100 (56.5)
Self-harm	3 (11.5)	6 (9.7)	4 (6.7)	2 (6.9)		15 (8.5)
Absconding	1 (3.8)	13 (21.0)	12 (20.0)	0		26 (14.7)
Damage to property	1 (3.8)	12 (19.4)	5 (8.2)	0		18 (10.2)
Weapon	26	62	60	30	132	310
In possession	8 (30.8)	8 (12.9)	12 (20.0)	4 (13.3)	9 (6.8)	37 (11.9)
Type of weapon	8	8	12	4	9	41
Near object	2 (25.0)	7 (87.5)	4 (33.3)	4 (100.0)	8 (88.9)	25 (61.0)
Other	6 (75.0)	1 (12.5)	8 (66.7)	0	1 (11.1)	16 (39.0)
Bystanders present	26	60	55	31		172
Yes	5 (19.2)	10 (16.7)	7 (12.7)	6 (19.4)		28 (16.3)
No	21 (80.8)	50 (83.3)	48 (87.3)	25 (80.6)		144 (83.7)
Injuries	26	62	60	29	132	309
Incidents where injury occurred	8 (30.8)	10 (16.1)	13 (21.7)	2 (6.9)	13 (9.8)	46 (14.9)
Injured person	8	11	13	2	13	47
Staff	7 (87.5)	4 (36.4)	13 (100.0)	1 (50.0)	12 (92.3)	37 (78.7)
Patient	1 (12.5)	5 (45.5)	0	0	1 (7.7)	7 (14.9)
Not specified	0	2 (18.2)	0	1 (50.0)	0	3 (6.4)
Management**	21	62	60	31	120	294
Sedation	3 (14.3)	26 (41.9)	39 (65.0)	5 (16.1)	61 (50.8)	134 (45.6)
Restraint	6 (28.6)	10 (16.1)	32 (53.3)	5 (16.1)	73 (60.8)	126 (42.9)
Deescalation	2 (9.5)	21 (33.9)	13 (21.7)	15 (48.4)	20 (16.7)	71 (24.1)
Escorted out	1 (4.8)	6 (9.7)	4 (6.7)	8 (25.8)	5 (4.2)	24 (8.2)
Security presence only	0	1 (1.6)	2 (3.3)	19 (61.3)	0	22 (7.5)

*Characteristics with totals less than 314 represent missing data.

**Categories are not mutually exclusive.

Verbal violence was the most common form of violence followed by physical violence. Weapons were used in 11.9% (*n* = 37) of incidents. Weapons were either convenient near objects or items specifically brought to the ED. Near objects included medical equipment (cardiac monitor, stethoscope, cervical collar, intravenous cannula); hot drinks and food; personal items (walking stick, mobile phone, clothing); furniture (table, sign, rubbish bin); broken glass; a live electrical wire; and a bottle of urine. Weapons brought to the ED included knives, razors, scissors, petrol, lighter, shotgun bullets, and trade tools such as crowbars, screwdrivers, screws, metal bars and a star picket.

Injuries to staff included emotional exhaustion and physical injuries ranging from soft tissue injuries, bruising, cuts, and skin tears to fractures and chest and back injuries. Other reported injuries included bites and exposure to body fluids (blood, saliva) including from spitting. Injuries to the perpetrator were almost always self-inflicted and included bites, cuts and skin tears, and partial-thickness burns.

**Figure 2.**
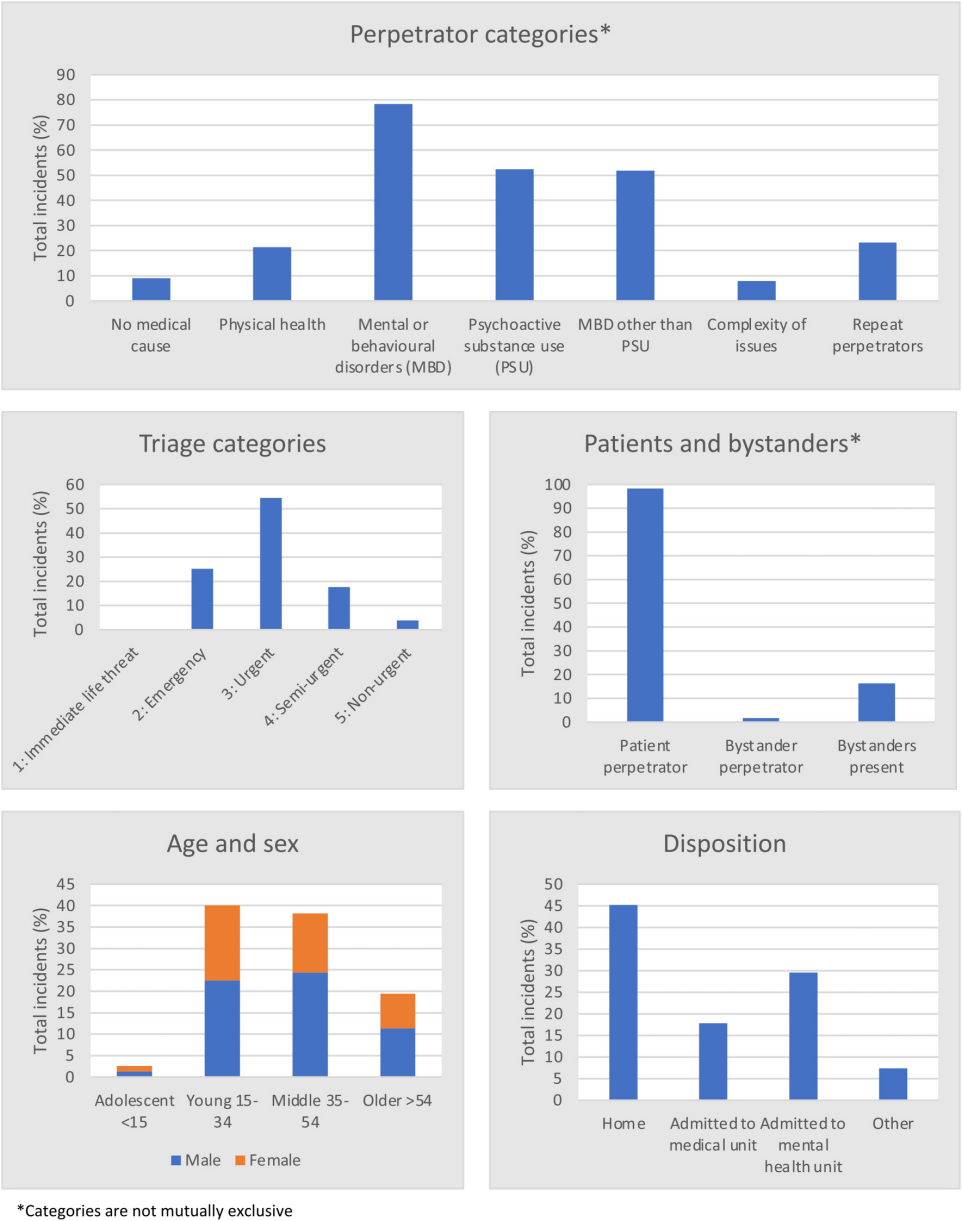
Perpetrator characteristics of violent incidents in regional and remote Australian EDs.

*Note.* ED = emergency department.

Perpetrator characteristics and categories from four of the five sites are presented in Figure 2 and include age, sex, triage category, and disposition. Perpetrators were predominantly young and middle-aged males and almost always patients being treated in the ED. The most common category of the perpetrator was those with psychoactive substance use, followed by a reported mental or behavioral disorder other than psychoactive substance use. A more comprehensive record of the perpetrator characteristics per site is in Table 2.

**Table 2.** Perpetrator Characteristics Summary.

	Site 1 *n* (%)	Site 2 *n* (%)	Site 3 *n* (%)	Site 4 *n* (%)	Total, n (%)
Age	21	60	52	28	161*
Adolescent <15	0	0	4 (7.7)	0	4 (2.5)
Young 15–34	6 (28.6)	27 (45.0)	23 (44.2)	8 (28.6)	64 (39.8)
Middle 35–54	10 (47.6)	25 (41.7)	14 (26.90	13 (46.4)	62 (38.5)
Older >54	5 (23.8)	8 (13.3)	11 (21.2)	7 (25.0)	31 (19.3)
Sex	23	61	56	29	169
Male	16 (69.6)	37 (60.7)	32 (57.1)	16 (55.2)	101 (59.8)
Female	7 (30.4)	24 (39.3)	24 (42.9)	13 (44.8)	68 (30.2)
Perpetrator category**	20	61	55	28	164
No medical cause	6 (30.0)	7 (11.5)	0 (0.0)	2 (7.1)	15 (9.1)
Physical health	1 (5.0)	7 (11.5)	13 (23.6)	14 (50.0)	35 (21.3)
Mental or behavioral disorders (MBD)	14 (70.0)	49 (80.3)	49 (89.1)	18 (64.3)	130 (78.3)
Psychoactive substance use (PSU)	6 (30.0)	31 (50.8)	33 (60.0)	16 (57.1)	86 (52.4)
MBD other than PSU	9 (45.0)	34 (55.7)	38 (69.1)	4 (14.3)	85 (51.8)
Complexity of issues	3 (15.0)	8 (13.1)	2 (3.6)	0	13 (7.9)
Repeat perpetrators	7 (35.0)	17 (27.9)	14 (25.5)	0	38 (23.2)
Triage category	11	51	43	26	131
Category 1: Immediate life threat	0	0	0	0	0
Category 2: Emergency	3 (27.3)	6 (11.8)	18 (41.9)	6 (23.1)	33 (25.2)
Category 3: Urgent	6 (54.5)	26 (51.0)	20 (46.5)	18 (69.2)	70 (54.4)
Category 4: Semiurgent	2 (18.2)	15 (29.4)	4 (9.3)	2 (7.7)	23 (17.6)
Category 5: Nonurgent	0	4 (7.8)	1 (2.3)	0	5 (3.8)
Time in ED prior to alert	9	53	56	26	144
<5 min	0	4 (7.5)	7 (12.5)	15 (57.7)	26 (18.1)
5–60 min	1 (11.1)	13 (24.5)	16 (28.6)	4 (15.4)	34 (23.6)
1–4 hr	4 (44.4)	22 (41.5)	16 (29.6)	4 (15.4)	46 (31.9)
4–12 hr	2 (22.2)	9 (17.0)	10 (17.9)	2 (7.7)	23 (16.0)
>12 hr	2 (22.2)	5 (9.4)	7 (12.5)	1 (3.8)	15 (10.4)
Ambulance	26	58	55	28	167
Arrival via ambulance	13 (50.0)	30 (51.7)	34 (61.8)	7 (25.0)	84 (50.3)
Issues with Paramedics	4 (30.8)	14 (46.7)	11 (32.3)	2 (28.6)	31 (36.9)
Police	26	59	53	27	165
Police involvement	9 (34.6)	33 (55.9)	29 (54.7)	7 (25.9)	78 (47.3)
Assessment order	20	61	51	23	155
	1 (5.0)	6 (9.8)	8 (15.7)	2 (8.7)	17 (11.0)
Psychiatric past history	20	61	60	29	170
Previous psychiatric diagnosis	11 (55.0)	50 (82.0)	42 (70.0)	5 (17.2)	110 (64.7)
Alert for violence in medical record	5 (25.0)	10 (16.4)	6 (10.0)	5 (17.2)	26 (15.3)
Drugs and alcohol	20	61	60	31	172
Either	6 (30.0)	31 (50.8)	33 (55.0)	16 (51.6)	86 (50.0)
Alcohol use	4 (20.0)	14 (23.0)	13 (21.3)	12 (38.7)	43 (25.0)
Drug use	4 (20.0)	18 (29.5)	27 (45.0)	4 (12.9)	53 (30.8)
Discharge	10	47	53	25	135
Home	8 (80.0)	19 (40.4)	18 (34.0)	16 (64.0)	61 (45.2)
Admitted to medical unit	0	10 (21.3)	8 (15.1)	6 (24.0)	24 (17.8)
Admitted to mental health unit	1 (10.0)	13 (27.7)	24 (45.3)	2 (8.0)	40 (29.6)
Other	1 (10.0)	5 (10.6)	3 (5.7)	1 (4.0)	10 (7.4)

*Characteristics with totals less than 181 represent missing data.

**Categories are not mutually exclusive.

## Discussion

The aim of this exploratory study was to identify the perpetrator and situational characteristics associated with violent incidents in the ED across five regional Australian sites. The discussion section begins with a summary of the notable findings and then moves on to a discussion on the nature and impact of violence in EDs, the approach to MBDs, and implications for the future of workplace violence research.

Violent incidents occurred relatively evenly throughout the week and across shift times. This is in contrast to the times for all ED presentations across Australia, with far fewer presentations on late and night shifts (Australian Institute of Health Welfare, 2020). The higher rate of violent incidents on late and night shifts compared to presentation times, in general, is comparable to the presentation time for patients with medication overdose, which are predominantly on late shifts (Buykx et al., 2010). The even spread of incidents across shift times may have implications for the rostering of staff and the availability of resources on late and night shifts. The incidence of violence was lower in this study than in other similar international studies, however, the rate of injuries was higher. The injuries were identified in these studies without specific definitions and via incident management systems, consistent with this study (James et al., 2006; Kleissl-Muir et al., 2018, 2019; Nikathil et al., 2017). Almost one in every six incidents resulted in an injury, which predominantly affected staff members. There were high rates of sedation and physical restraint.

### Nature of Violence in ED

Workplace violence has an insidious nature and can occur in many ways, shapes and forms (Spelten et al., 2020b). There were several acts of extreme physical violence noted in this study, resulting in fractures and lacerations to staff, but many more instances of less severe violence which still impact the workers involved. Workplace violence is more diverse still, with violence from patients directed at themselves, and the profound impact this may have on the patient, staff, and bystanders.

### Emerging Recognition of Nonphysical Aggression

The exposure to nonphysical violence within the ED appears to be constant, unavoidable, and dangerous (Spelten et al., 2020a, 2020b; Thomas et al., 2020). There is growing awareness of the significant impact that long-term repetition of nonphysical violence has on people, and the need to highlight that behavior (Hannem et al., 2015). In healthcare, particularly in regional and remote areas, staff may be required to maintain extended therapeutic relationships with patients who behave aggressively, without being physically violent (Jacob et al., 2020). Exposure to verbal abuse and threats has been linked to higher levels of stress and lower levels of psychological well-being among healthcare staff (Lee & Lee, 2022). This understanding of the impact of nonphysical violence should be acknowledged and applied to the experience of healthcare workers.

In addition to the direct outcomes of workplace violence, indirect outcomes include reduced staff satisfaction and staff retention (Schat & Kelloway, 2003). Staff retention is one of the most important issues facing healthcare organizations worldwide and has a significant impact on EDs which are resource-intensive (Mohammad & Chagani, 2015; Schat & Kelloway, 2003). This is especially significant in regional and remote settings where workforce shortages are more pronounced (Australian College of Nursing, 2018; Australian Institute of Health Welfare, 2019b). Organizational support and follow-up have been associated with a moderating effect on the emotional well-being and resilience of assaulted staff, and improvements in job satisfaction, burnout, turnover intention and quality of care (Schat & Kelloway, 2003; Thomas et al., 2021). This follow-up with assaulted staff should occur in the immediate response to an incident, in the incident investigation, and in long-term support (Friis et al., 2019; Thomas et al., 2021).

### Mental and Behavioral Disorders

Across all sites with available perpetrator data, the majority of incidents involved a perpetrator with a mental health or psychoactive substance use diagnosis. The pressure of increasing demand for mental health services coupled with a lack of adequate community-based services means that EDs are increasingly being accessed by people requiring mental health care (Australasian College for Emergency Medicine, 2019). Compared to individuals with other emergency medical conditions, those experiencing an acute mental health or behavioral crisis are 16 times more likely to arrive with police or correctional services, almost twice as likely to arrive via ambulance or rescue services and twice as likely to leave the ED before their treatment has finished (Australasian College for Emergency Medicine, 2018). Managing violent behavior in this cohort of patients is resource-intensive and carries implications for the resource management of EDs (Oliver et al., 2019).

In order to reduce unnecessary ED presentations for MBDs, several non-ED initiatives have been put in place. Collaborative programs combining mental health clinicians with police and/or paramedics to divert patients away from EDs have become popular across Australia (Park et al., 2021; Puntis et al., 2018). These multiagency interventions provide early deescalation and provide alternative pathways to care leading to a reduction in ED presentations (Henry & Rajakaruna, 2018; Park et al., 2021; Puntis et al., 2018). They have been associated with up to 80% reduction in transfers to the ED (ABC News, 2020; Henry & Rajakaruna, 2018), and are reported to be invaluable and a necessary interim solution for a lack of community mental health support, however are at risk without continued commitment and funding from government (ABC News, 2020; Parliament of Victoria, 2019). Innovative programs such as these may be useful in regional and remote areas to link people with mental health crises to alternate services and prevent unnecessary ED presentations.

There is a cohort of patients with MBDs that require the ED, in these situations, behavioral assessment rooms (BAR) or units (BAU) are recommended to remove these patients from the chaotic and overstimulating environment of the ED (Braitberg et al., 2018; Department of Health and Human Services, 2017; Weiland et al., 2017). BAR/BAUs located within an ED have been associated with lower incidence of security alerts, mechanical restraint and therapeutic sedation as well as decreases in ED length of stay, however, this is preliminary evidence and controlled trials are required to determine their effectiveness (Braitberg et al., 2018; Weiland et al., 2017)

### Future of Violence Prevention

The diverse reporting procedures across health services encountered while conducting this study highlight the difficulty in gaining timely access to comparable, high-quality data. This has implications for routine monitoring and for evaluating the impact of any policy interventions to reduce violence, particularly across jurisdictions (Doyle, 2015). Work needs to be done to standardize practice regarding reporting of workplace violence in healthcare. Previous research has shown that up to 88% of people who experience workplace violence do not call a Code Black event or document this in patient notes (Arnetz et al., 2015). A potential benefit of such standardization would be the ability to more reliably evaluate the effectiveness of interventions (Murray et al., 2020; Spelten et al., 2020a). The approach to violence prevention in Australia has been reported to be disjointed, with systemic failures in policies and procedures to prevent workplace violence in healthcare as well as failures of agencies to fulfill the expectations outlined in the policies (Doyle, 2015). This is in complete contrast to the national approach to patient safety in hospitals where all health services are required to be assessed and accredited to a single national standard (Australian Commission on Safety and Quality in Health Care, 2021). A national standard may help in processes to protect staff being afforded the same importance as processes to protect patients, and further, standardized practices may help prompt policy development and effective enforcement. Priorities for regional and remote EDs include addressing the everyday abuse staff are exposed to and supporting those staff in the short and long-term to moderate the impact on attrition in an already understaffed workforce. Efficiencies in finding somewhere else for patients to go outside of the ED when appropriate is also essential for regional and remote EDs to avoid the increased burden on limited resources and services.

## Limitations

The likely underreporting of incidents is a limitation of this study. As workplace violence is highly complex, the antecedents to violent incidents are numerous, many are not recorded in the emergency healthcare setting and therefore could not be collected. The proportion of “serious” incidents (i.e., those involving weapons and/or injuries) relative to overall incidents reported varied greatly between sites. The reasons for this difference are not clear and could reflect true differences, differences in reporting practices, or some other factor. Perpetrator-specific data was only available from four sites, although those four sites represented diverse coverage of EDs in regional and remote areas of Australia. Another limitation is the number of violent incidents did not allow statistical comparisons across sites. The data analysis is descriptive and not comparative or correlational. The diverse reporting procedures across health services and variability in quality and ease of access to data should be noted for improvement at a policy level.

## Implications for Practice

Knowing the characteristics of perpetrators of workplace violence can help in seeking to tailor interventions to reduce further violent behaviors. This study in rural and regional Australian EDs found that perpetrators of violence were predominantly young and middle-aged males and almost always a patient, with most presenting with MBDs, or PSU. Targeting future interventions to reduce aggression in this group may increase the likelihood of positive responses. There is a need for interdisciplinary collaboration with organizations external to the hospital setting, and a focus on understanding and management of mental health and intoxication.

## Conclusion

Workplace violence has an insidious and destructive nature and can manifest in many ways. Greater awareness of the impact and approach to addressing nonphysical violence is required. Data gathered for this study from regional EDs paints a similar picture to that of metropolitan EDs. The majority of violent incidents involve a patient with mental health issues or psychoactive substance use. Managing violent behavior in this cohort of patients appears resource intensive, often requiring sedation and restraint of perpetrators and multiagency collaboration, this may carry implications for the resource management of EDs, especially in regional and remote areas and warrants future research into targeted interventions for these categories of perpetrators. There is difficulty in gaining timely access to comparable, high-quality data across health services and jurisdictions. This has implications for routine monitoring and for evaluating interventions to reduce violence.
